# Proteomic analysis of α4β1 integrin adhesion complexes reveals α-subunit-dependent protein recruitment

**DOI:** 10.1002/pmic.201100487

**Published:** 2012-07-17

**Authors:** Adam Byron, Jonathan D Humphries, Sue E Craig, David Knight, Martin J Humphries

**Affiliations:** 1 Wellcome Trust Centre for Cell-Matrix Research, Faculty of Life Sciences, University of ManchesterManchester, UK; 2 Biological Mass Spectrometry Core Facility, Faculty of Life Sciences, University of ManchesterManchester, UK

**Keywords:** Adhesion complexes, Cell adhesion, Cell biology, Integrin, Signaling

## Abstract

Integrin adhesion receptors mediate cell–cell and cell–extracellular matrix interactions, which control cell morphology and migration, differentiation, and tissue integrity. Integrins recruit multimolecular adhesion complexes to their cytoplasmic domains, which provide structural and mechanosensitive signaling connections between the extracellular and intracellular milieux. The different functions of specific integrin heterodimers, such as α4β1 and α5β1, have been attributed to distinct signal transduction mechanisms that are initiated by selective recruitment of adhesion complex components to integrin cytoplasmic tails. Here, we report the isolation of ligand-induced adhesion complexes associated with wild-type α4β1 integrin, an activated α4β1 variant in the absence of the α cytoplasmic domain (X4C0), and a chimeric α4β1 variant with α5 leg and cytoplasmic domains (α4Pα5L), and the cataloguing of their proteomes by MS. Using hierarchical clustering and interaction network analyses, we detail the differential recruitment of proteins and highlight enrichment patterns of proteins to distinct adhesion complexes. We identify previously unreported components of integrin adhesion complexes and observe receptor-specific enrichment of molecules with previously reported links to cell migration and cell signaling processes. Furthermore, we demonstrate colocalization of MYO18A with active integrin in migrating cells. These datasets provide a resource for future studies of integrin receptor-specific signaling events.

Adhesion to the extracellular matrix (ECM) and to adjacent cells is essential for a multicellular existence. A major family of cell adhesion receptors are the integrins, which are noncovalently associated αβ heterodimers. The selective pairing of 18 α and 8 β subunits results in 24 heterodimer combinations [1]. Many ECM and cell-surface adhesion molecules bind to multiple integrins, and most integrin receptors recognize a wide variety of ligands [2]. The particular integrin–ligand combination elicits a specific signaling response that is transduced across the plasma membrane by integrins; however, a comprehensive understanding of ligand-specific integrin signaling is currently lacking. In addition to ligand-induced signaling, integrins can be primed for ligand binding and activated into a high-affinity conformation by the binding of molecules to the cytoplasmic tails of integrins. Interactions between α and β integrin cytoplasmic domains regulate integrin activation [3–7]. The cytoskeletal molecule talin, which binds β integrin cytoplasmic tails, plays a key role in integrin activation by preventing the interaction between α and β integrin tails [6, 8]. Furthermore, the cytoplasmic domains of specific integrins determine functional differences between integrin heterodimers [9–12]. For effective signaling, integrin activity must by tightly controlled, and dysregulation of adhesion signaling plays key roles in many disease states, including dysregulated hemostasis, inflammation, fibrosis, and cancer.

Integrin signal transduction is mediated by membrane-associated adhesion complexes and mechanosensitive connections to the actin cytoskeleton [13, 14]. The molecular composition of adhesion signaling complexes plays a central role in determining their function, although – like other membrane receptor-associated signaling complexes – integrin adhesion complexes have been difficult to analyze by proteomic techniques due to their lability and inaccessibility. This refractoriness is largely due to (1) the low-affinity, mechanosensitive protein–protein interactions that enable dynamic signaling and (2) their location at the plasma membrane, linking cytoskeletal molecules to transmembrane receptors and extracellular ligands, which renders them difficult to solubilize. We have developed a methodology for the affinity isolation and mass spectrometric analysis of stabilized, ligand-induced integrin adhesion complexes [15, 16]. Here, we used this approach to investigate the recruitment of integrin heterodimer-specific signaling complexes. Specifically, we examined the role of the α4 integrin cytoplasmic domain in recruiting specific components to adhesion complexes. α4β1 integrin is expressed on a variety of blood cells, and its prototypic counter-receptor ligand vascular cell adhesion molecule-1 (VCAM-1) reflects its involvement in adhesion to the vascular endothelium during chronic inflammatory diseases, such as psoriasis, rheumatoid arthritis, asthma, and allergy [17]. α4β1 integrin expression is also characteristic of advanced primary tumors, and it mediates the interaction of cancer cells with VCAM-1, e.g. myeloma cells with VCAM-1 on bone marrow stroma [18].

Integrin activation is coupled to spatial separation of the cytoplasmic domains of the integrin heterodimer [7]. To examine the recruitment of proteins to adhesion complexes in the absence of β integrin tail interactions with an α integrin tail, we used an α4 integrin C-terminal cytoplasmic domain deletion mutant (X4C0) (Fig. 1A) [19]. This mutant may mimic the cytoplasmic domain separation linked to integrin activation because of the loss of the α–β clasp, the loss of lateral association with other cell-surface receptors, the exposure of β1 integrin-binding sites or the reduced steric hindrance around the β1 integrin tail in the absence of the α4 integrin tail. The truncation of the α4 cytoplasmic domain has been used previously to indicate a role for α4 integrin in a cell motility signaling pathway that is distinct from α5 integrin-stimulated migration [9, 12]. To examine differences in recruitment of proteins to α4β1 and α5β1 integrin cytoplasmic domains, we used an α4α5 integrin chimera comprising the N-terminal β-propeller domain of α4 integrin – which determines ligand specificity – linked to the leg, transmembrane and cytoplasmic domains of α5 integrin (α4Pα5L) (Fig. 1A) [20]. K562 human chronic myelogenous leukemia cells were used for our work because this cell line has served as a prototype for many analyses of integrin signaling and adhesion. The integrin complement of K562 cells is dominated by α5β1 integrin, which enabled us to use cells into which wild-type (WT) α4 integrin, X4C0, or α4Pα5L transgenes had been introduced. These cell lines are referred to here as K562-α4, K562-X4C0 (gifts from M. E. Hemler, Dana-Farber Cancer Institute, Boston, MA, USA) [19], and K562-α4Pα5L [20], respectively. All cell lines were cultured in RPMI 1640 medium supplemented with 10% (v/v) FCS (Lonza Bioscience, Wokingham, UK), 2 mM l-glutamine, and Geneticin (1 mg/mL; Invitrogen, Paisley, UK) at 37°C.

**Figure 1:**
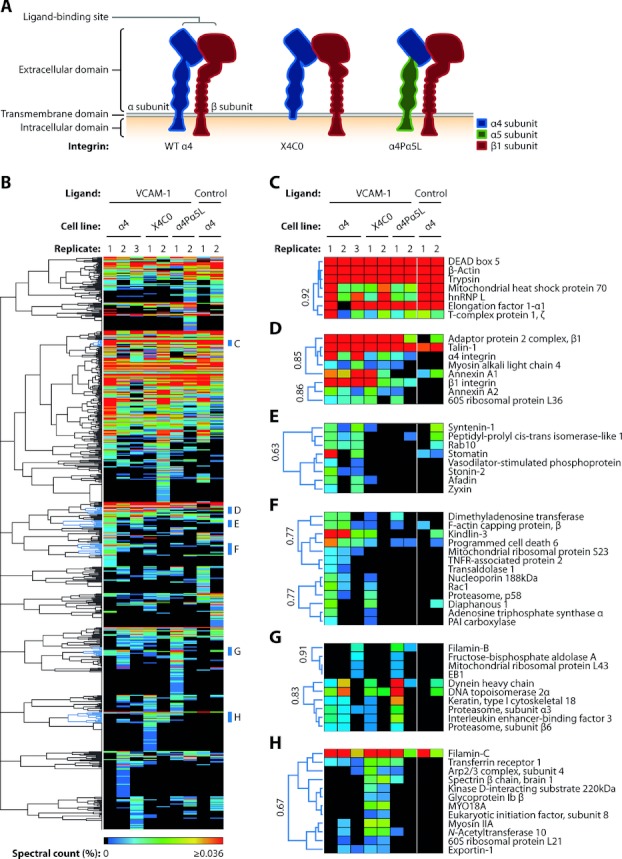
Hierarchical clustering analysis of proteins identified by MS ana-lyses of VCAM-1 and control affinity purifications. (A) Integrin-associated complexes were isolated from K562 cells expressing WT α4 integrin (α4), α4 integrin with a cytoplasmic domain deletion (X4C0), and an α4α5 chimera comprising the N-terminal β-propeller domain of α4 integrin linked to the leg, transmembrane, and cytoplasmic domains of α5 integrin (α4Pα5L). (B) Complete output of hierarchical clustering analysis of identified proteins (see Supporting Information for details). (C**–**H) Selected clusters are displayed, as indicated by blue dendrogram nodes and bars in (B). Correlations at selected dendrogram nodes are indicated. PAI, phosphoribosylaminoimidazole; TNFR, tumor necrosis factor receptor.

Plasma membrane fractions enriched for integrin membrane complexes were isolated using the ligand affinity purification approach described by Humphries et al. [15] as described in the Supporting Information. As a ligand for α4β1 integrin, we used VCAM-1, and as a nonintegrin-binding control, we used mutant VCAM-1(D40A) that contains an aspartate-40--alanine point mutation in the integrin-binding site. Recombinant soluble VCAM-1 and VCAM-1(D40A) were expressed from the pIG1 vector and purified as described previously [21]. Paramagnetic beads (Invitrogen, Paisley, UK) coated with VCAM-1 were incubated with K562 cell lines to induce the formation of integrin adhesion complexes in living cells. After 30-min incubation of cells with VCAM-1-coated beads, labile integrin-associated complexes were stabilized with 3 mM dimethyl-3,3′-dithiobispropionimidate (Thermo Fisher Scientific, Rockford, IL, USA), a membrane-permeable, thiol-cleavable cross-linker. Bulk cell membrane was removed using detergent lysis and sonication (see Supporting Information for details), which permitted the enrichment of α4β1 integrin and adhesion complex components on integrin ligand-coated beads as compared with control beads (Supporting Information Fig. S1) [15, 16].

We used MS-based proteomics to catalogue the components of the isolated integrin adhesion complexes. MS data acquisition and analysis were performed as described in the Supporting Information. MS analysis identified in total 642 unique proteins with a probability of at least 99% and at least two unique peptides, with an estimated protein false discovery rate of 0.1%. Data were acquired from two biological replicate experiments (three for VCAM-1 isolations from K562-α4 cells). Relative protein abundance was determined by spectral counting. The enrichment of adhesion complex components strongly and weakly enriched to VCAM-1 compared to control beads as determined by spectral counting was validated by immunoblotting (Supporting Information Fig. S1). MS data were converted using PRIDE Converter (version 2.5.3) [22] and deposited in the PRIDE database (http://www.ebi.ac.uk/pride) [23] under accession numbers 19 284–19 301. Details of all identified proteins and peptides are provided in the Supporting Information Tables S1 and S2.

As an important validation of the specificity of these data, we confirmed that α4 integrin peptides detected in samples from K562-α4 cells matched to sequences throughout the full length of WT α4 integrin, including the C-terminal cytoplasmic domain, whereas α4 integrin peptides detected in samples from K562-X4C0 cells matched to α4 integrin sequences that excluded the C-terminus absent from the X4C0 cytoplasmic domain deletion mutant (Supporting Information Table S2). Also, in samples from K562α4Pα5L cells, detected α4 integrin peptides matched to sequences restricted to the N-terminal β-propeller domain of α4 integrin, whereas α5 integrin peptides only matched to sequences in the C-terminal leg region of α5 integrin that was present in the α4α5 chimera (Supporting Information Table S2). No peptides matching to the leg region of α4 integrin were detected in samples from K562-α4Pα5L cells, and no α5 peptides were detected in samples other than those from K562-α4Pα5L cells. Moreover, no integrin peptides were detected in the nonintegrin-binding control. These data therefore indicate the isolation of distinct integrins from each cell line and support the isolation of specific integrin-associated complexes.

To aid the interrogation and visualization of the MS datasets, and to enable hypothesis generation for future experimental testing, hierarchical clustering analysis was performed on the quantitative data (Fig. 1, B–H). Hierarchical clustering analysis organized proteins according to the similarity of their enrichment profiles across the different samples, adjoining proteins with similar enrichment profiles into groups (clusters; see Supporting Information for details). Clustering using a Pearson correlation-based distance metric identified clusters of proteins (including selected clusters C–H) enriched in different VCAM-1-induced adhesion complexes (Fig. 1B). Based on the pattern of protein enrichment, several nonspecific or reagent proteins, such as mitochondrial heat shock protein 70 and trypsin, were grouped in one of several putative “background” clusters, exemplified by cluster C (Fig. 1C). We hypothesized that these clusters represented nonspecific proteins because the enrichment profiles were dominated by strong detection in the control samples and no relative enrichment in the VCAM-1 samples. To highlight proteins with distinct enrichment profiles, we selected several clusters of interest based on distinct protein enrichment using different cell lines, ensuring a Pearson correlation threshold of at least 0.6 and absence or low detection in the control samples (see Supporting Information for details). Two adjacent subclusters of proteins (forming cluster D) were strongly enriched in VCAM-1 affinity purifications using all cell lines as compared to control purifications (Fig. 1D). This cluster included α4 and β1 integrins and the integrin-binding protein talin-1, which are known to associate in the same protein complexes, thus demonstrating the relevance of hierarchical clustering analysis for interrogation of these data. Talin-1 was strongly enriched by VCAM-1-coated beads as compared to control beads (8.5-fold) using K562-α4 cells, moderately enriched (4.1-fold) using K562-X4C0 cells and weakly enriched (1.9-fold) using K562-α4Pα5L cells (Supporting Information Table S3). Another cluster comprising proteins mainly isolated in VCAM-1 affinity purifications using K562-α4 cells (cluster E) included proteins that have previously been shown to have roles in cell adhesion, such as afadin, vasodilator-stimulated phosphoprotein, and zyxin, and proteins with roles in endocytosis, such as Rab10, stomatin, and stonin-2 (Fig. 1E). Two adjacent subclusters of proteins that were largely enriched using K562-α4 and K562-X4C0 cells (cluster F) included proteins with known functions in cell adhesion, such as kindlin-3, diaphanous 1, and Rac1. These subclusters also contained proteins not known to play a role in cell adhesion but with functions in cell signaling, such as programed cell death 6 and tumor necrosis factor receptor-associated protein 2 (Fig. 1F). Tumor necrosis factor receptor-associated protein 2 has been reported to interact with filamin-A to regulate inflammatory signal transduction [24], but may, on the basis of the data presented here, also play a role in integrin signaling. Cluster G contained proteins that were enriched using K562-α4Pα5L cells, e.g. the microtubule tip-binding proteins EB1 and dynein heavy chain (Fig. 1G). Cluster H revealed that the cytoskeletal components actin-related protein (Arp) 2/3 complex subunit 4, filamin-C, myosin IIA, MYO18A, and spectrin β chain were enriched using K562-X4C0 cells (Fig. 1H). It has been reported that the lack of an α integrin cytoplasmic domain results in minimal cell migration [25], which may reflect more stable links to the cytoskeleton suggested by these data. Filamin-C was enriched by VCAM-1-coated beads as compared to control beads (4.3-fold) using K562-X4C0 cells, but not using K562-α4 or K562-α4Pα5L cells (Supporting Information Table S3). In contrast, peptides specific to filamin-B were only detected using K562-α4 or K562-α4Pα5L cells, but not using K562-X4C0 cells, which suggests that different filamins were recruited preferentially to different integrin heterodimers.

To examine the molecular landscape of the isolated integrin adhesion complexes in the context of currently known protein–protein interactions, and as a complementary approach to hierarchical clustering analysis, interaction network analysis was used. We generated a human interactome consisting of physical protein–protein interactions reported in multiple databases (see Supporting Information for details). We mapped onto this interactome 338 (97%) of the 350 proteins enriched in the VCAM-1 affinity purifications. A threshold of one standard deviation greater than the mean enrichment ratio (VCAM-1 over control) was used as a cut-off for specific enrichment in VCAM-1 affinity purifications, which equated to a 1.82-fold change (Supporting Information Fig. S2). To generate a focused dataset, we extracted all proteins reported in a literature-curated database of adhesion-related components (the “adhesome”) [26] and displayed them as an interaction network (Fig. 2). The proteins (nodes) were annotated to indicate their subcellular localization according to the Gene Ontology (GO) database (release date 28th January 2012) [27]. Almost all proteins in the network were annotated as cytoplasmic, as expected for proteins in a complex associated with the cytoplasmic tails of integrins, and 22 (73%) out of 30 proteins were reported by GO annotation as transmembrane or cell periphery components (e.g. plasma membrane-associated or focal adhesion components). The interaction network was arranged as sets of proteins that were identified using one or multiple cell lines. The overlaps of these sets highlight distinct heterodimer-dependent protein recruitment. As discussed above, α4 and β1 integrins (*ITGA4* and *ITGB1*, respectively) were enriched in all cell lines, and are displayed in the central intersection of all three sets in the network (Fig. 2). Also present in this intersection set are the following proteins: talin-1 (*TLN1*), a key activator of integrin function; moesin (*MSN*), a member of a family of plasma membrane-actin cytoskeleton linker proteins; subunit 4 of the Arp2/3 complex (*ARPC4*), which controls actin polymerization; and adenosine 5′-diphosphate ribosylation factor (Arf) 1 (*ARF1*), a small guanosine triphosphatase (GTPase) that plays a role in vesicular trafficking. In the context of the adhesome database, these proteins represent a “core” set that is associated with all three types of integrin tested in this study, which implicates several major functions of cell adhesion: integrin activation, cytoskeletal linkage, regulation of the cytoskeleton, and protein trafficking.

**Figure 2:**
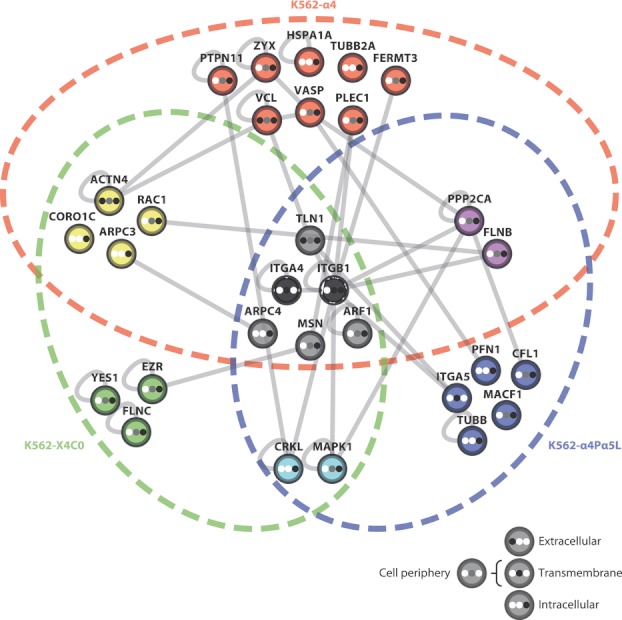
Interaction network analysis of adhesome components enriched in VCAM-1-induced adhesion complexes. Specifically enriched proteins (enriched at least 1.82-fold compared to the control; Supporting Information Fig. S2) reported as adhesome components by Zaidel-Bar and Geiger [26] were visualized as an interaction network. Of the 174 adhesome components, we identified 26 (15%) in our datasets, although 30 proteins are displayed in the interaction network due to the detection of multiple subunits or paralogs. Nodes (circles) represent identified proteins and are labeled with gene symbols; edges (gray lines) indicate reported protein–protein interactions. Nodes are clustered and colored according to their enrichment using one or multiple cell lines; the Venn diagram sets (dashed lines) indicate using which cell line each protein was enriched: red, K562-α4; green, K562-X4C0; blue, K562-α4Pα5L. Nodes contain dots that indicate subcellular localization of proteins according to GO annotation with cellular component terms. The parent terms used (derived from which all child terms were also considered) were as follows: extracellular, GO:0005576 (extracellular region); cell periphery, GO:0009986 (cell surface), GO:0030054 (cell junction), and GO:0071944 (cell periphery); transmembrane, GO:0031226 (intrinsic to plasma membrane); intracellular, GO:0005622 (intracellular).

In addition to core components, many subunit-specific proteins were detected. In support of the observation using clustering that some tubulin-binding proteins were enriched using K562-α4Pα5L cells (Fig. 1G), interaction network analysis showed that microtubule-actin cross-linking factor 1 (*MACF1*, also known as ACF7) was also enriched using K562-α4Pα5L cells (Fig. 2). A broader interaction network analysis in the context of all reported protein–protein interactions was performed to enable a less restricted interrogation of the datasets (Supporting Information Fig. S3). GO enrichment analysis of the sets of proteins recruited to each integrin heterodimer revealed overrepresentation of the term “focal adhesion” in all subnetworks (KEGG term hsa04510; fold enrichment >5; Bonferroni-corrected *p*-value < 0.1), indicating a “core” of adhesion molecules present in all complexes. Analysis of subnetworks isolated using K562-X4C0 or K562-α4Pα5L cells showed overrepresentation of the term “regulation of actin cytoskeleton” (KEGG term hsa04810; fold enrichment > 6; Bonferroni-corrected *p*-value < 0.01), which supports our hierarchical clustering analysis (Fig. 1, G and H). Analysis of subnetworks isolated using K562-α4 or K562-X4C0 cells revealed overrepresentation of the term “leukocyte transendothelial migration” (KEGG term hsa04670; fold enrichment > 11; Bonferroni-corrected *p*-value < 0.001), which was accounted for by specific enrichment of term members such as α-actinin-4 (*ACTN4*), Rac1 (*RAC1*), and afadin (*MLLT4*) to WT α4 and X4C0 integrin heterodimers (Supporting Information Fig. S3). These data show that there is overrepresentation of overlapping and distinct terms in different subnetworks of the interaction network, which suggests that complexes recruited to specific integrin heterodimers may possess distinct functional repertoires. In addition to identifying the proteins highlighted by clustering and adhesome network analysis, interrogation of the broader interaction network revealed that the small GTPase Arf-like 2 (*ARL2*), which binds microtubules and regulates microtubule dynamics [28], was enriched using K562-α4Pα5L cells. In adenocarcinoma cells, Arf-like 2 regulates the localization and activity of the tumor suppressor protein phosphatase 2A (*PPP2CA*), which was enriched using K562-α4Pα5L and K562-α4 cells (Supporting Information Fig. S3). In addition to microtubule-associated proteins, proteins involved in the positive and negative regulation of actin filament growth (profilin-1, *PFN1*, and cofilin-1, *CFL1*, respectively), a process crucial for the generation of intracellular force and effective cell migration, were enriched using K562-α4Pα5L cells. These data suggest that control of cytoskeletal dynamics may be important for integrin heterodimer-specific differences in cell contractile forces and migration [9, 10, 12].

Interaction network analysis highlighted that the small GTPase Rac1, which drives adhesion initiation, was enriched using K562-α4 and K562-X4C0 cells but not using K562α4Pα5L cells (Fig. 2). Rac1 was clustered with other adhesion-related components, such as kindlin-3 and diaphanous 1, using hierarchical clustering analysis (Fig. 1F). Interestingly, we have shown previously that Rac1 was enriched to VCAM-1-induced α4β1 integrin as compared to fibronectin-induced α5β1 integrin complexes [15]. Our previous work also showed that regulator of chromosome condensation–2 (RCC2) was enriched in fibronectin–α5β1 integrin complexes and that RCC2 limited the activity of Rac1 and regulated fibronectin-dependent adhesion formation and directional cell migration [15]. Indeed, in this study, interaction network analysis revealed enrichment of RCC2 using K562-α4Pα5L cells but not using K562-α4 and K562-X4C0 cells (Supporting Information Fig. S3). Together, these data suggest that the differential recruitment of Rac1 and RCC2 to adhesion complexes represents a distinct, α-subunit-dependent mode of adhesion signaling that may play a role in integrin-specific cell migration [9, 10, 12, 15, 16].

We performed a comparative analysis of the proteins identified in adhesion complexes in this study and those identified in our previously reported proteomic analysis of adhesion complexes [15]. Notably, there was a considerable overlap between the proteins specifically enriched to VCAM-1 using K562-α4 cells in this study and using K562-α4 cells in our previous work (Supporting Information Fig. S4), and the scales of the proteomes were very similar (210 and 185 proteins, respectively). Indeed, of the 185 proteins enriched to VCAM-1 using K562-α4 cells in our previous work, 158 (85%) were enriched using K562-α4 cells in this study. In contrast, of the 406 proteins enriched to fibronectin using K562-α4 cells in our previous work, 133 (33%) were enriched using K562-α4 cells in this study (Supporting Information Fig. S4), indicating a similar number of components in common with either ligand-induced complex but highlighting the more substantial nature of the fibronectin-induced complex, perhaps reflecting engagement of multiple cell-surface receptors, and increased signaling agonism by fibronectin. Unlike using K562-α4 cells, proteins enriched using K562-X4C0 and K562-α4Pα5L cells overlapped less well with proteins enriched in previously reported VCAM-1 complexes (81 and 79 proteins, 44% and 43%, respectively, of the 185 proteins enriched to VCAM-1 using K562-α4 cells in our previous work) (Supporting Information Fig. S4). These data suggest that there is considerable reproducibility between independent isolations of like adhesion complexes using the methods described here and that different “flavors” of adhesion complex are recruited to specific integrin heterodimers.

To demonstrate the relevance of the data to adhesion biology, and as proof-of-principle that the datasets provide a resource that can be mined to guide future investigations, we validated the subcellular localization of a novel adhesion complex component identified in this study. We noted above that actomyosin components were enriched using K562-X4C0 cells (Fig. 1H). Several myosin chains were enriched using K562-X4C0 cells (Supporting Information Fig. S5), but none were identified in the interaction network analysis, possibly due to uncharacterized protein–protein interactions that are not present in the interactome. MYO18A (also known as myosin XVIIIA, MysPDZ, SPR210, or MAJN), which has not been previously reported in adhesion complexes, was only enriched using K562-X4C0 cells. To investigate the association of MYO18A in the context of active integrin, which the X4C0 molecule mimics, we transfected green fluorescent protein (GFP)–MYO18A (gift from K. Sutoh, University of Tokyo, Tokyo, Japan) into B16-F10 mouse melanoma cells, which express α4β1 integrin and are an established model for cancer metastasis, a process in which active integrin plays a role. An unconventional myosin, MYO18A has a PDZ scaffold module and an ATP-insensitive actin-binding site [29]. B16-F10 cells spread on VCAM-1 formed actin stress fibers and, as expected, we observed colocalization of MYO18A with F-actin by immunofluorescence (Fig. 3) [29, 30]. Interestingly, using an activation-specific anti-integrin monoclonal antibody, we observed colocalization of MYO18A and F-actin with active β1 integrin at membrane ruffles, which are characteristic features of many migrating cells (Fig. 3A). At putative retraction fibers at the cell trailing edge, MYO18A colocalized with F-actin that was extending to an integrin plaque, but MYO18A staining did not overlap with that of active β1 integrin, suggesting a distinct but connected localization along these cytoskeletal structures (Fig. 3B). MYO18A has been shown to localize to membrane ruffles in epidermoid carcinoma cells, where it binds to p21-activated kinase (PAK)-2 through the PAK-interacting exchange factor-G-protein-coupled receptor kinase-interactor-1 complex to regulate membrane ruffle formation, adhesion complex turnover, actin cytoskeleton reorganization, and cell migration [31]. Furthermore, a recent study in B cells identified MYO18A as a binding partner for ezrin [32]. As for MYO18A, ezrin (EZR) was specifically enriched using K562-X4C0 cells in this study (Fig. 2). The role of ezrin in linking the plasma membrane to the actin cytoskeleton raises the intriguing possibility that ezrin may connect MYO18A to sites of integrin adhesion. Thus, MYO18A may play an important role in mediating cell migration through association with integrin heterodimer-specific adhesion complexes. Understanding the exact contribution of these molecules to cell adhesion processes requires further investigation.

**Figure 3:**
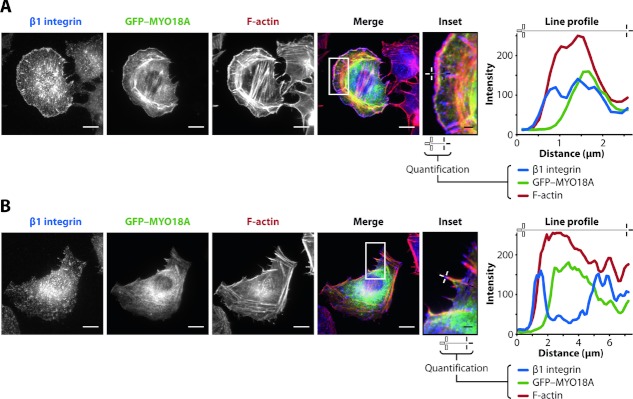
Localization of MYO18A to membrane ruffles and actin cytoskeleton proximal to integrin. (A and B) B16-F10 cells were transfected with GFP–MYO18A, spread on VCAM-1 for 60 min, fixed and stained for active β1 integrin (9EG7, which reports extended conformations of β1 integrin that occur upon ligand binding and integrin clustering in adhesion complexes [33]; blue), F-actin (phalloidin; red) and GFP (green). Insets detail the leading edge (A) and trailing edge (B) of cells (white box in merged images). Crosshairs in insets delimit lines along which pixel intensities were quantified in associated line profiles. Scale bars, 10 μm; except insets (black scale bars), 2 μm.

In summary, our MS datasets support the hypothesis of differential recruitment of proteins to specific integrin heterodimers in response to ligand binding. We used hierarchical clustering and interaction network analyses to assess the enrichment patterns of proteins to distinct signaling complexes. We identified previously unreported components of integrin adhesion complexes, observed the colocalization of MYO18A with active integrin in membrane ruffles of cells, and described receptor-specific enrichment of molecules associated with cell migration, signaling, and other cellular processes. Thus, we demonstrate that the catalogues of the adhesion complex proteomes and the data interrogation by clustering and network analyses, which are available as Supporting Information for further mining, can be effectively combined to identify new adhesion complex components with relevance to cell adhesion and migration. These datasets provide a valuable resource to aid discovery and further study of integrin receptor-specific signaling events.

## Abbreviations

Arf: adenosine 5′-diphosphate ribosylation factor
Arp: actin-related protein
ECM: extracellular matrix
GFP: green fluorescent protein
GO: Gene Ontology
GTPase: guanosine triphosphatase
PAK: p21-activated kinase
VCAM-1: vascular cell adhesion molecule–1
WT: wild-type

## Supplementary material

Disclaimer: Supplementary materials have been peer-reviewed but not copyedited.
